# Yeast-Derived Recombinant Avenanthramides Inhibit Proliferation, Migration and Epithelial Mesenchymal Transition of Colon Cancer Cells

**DOI:** 10.3390/nu10091159

**Published:** 2018-08-24

**Authors:** Federica Finetti, Andrea Moglia, Irene Schiavo, Sandra Donnini, Giovanni Nicolao Berta, Federica Di Scipio, Andrea Perrelli, Claudia Fornelli, Lorenza Trabalzini, Saverio Francesco Retta

**Affiliations:** 1 Department of Biotechnology, Chemistry and Pharmacy, University of Siena, 53100 Siena, Italy; finetti2@unisi.it (F.F.); schiavoirene@gmail.com (I.S.); lorenza.trabalzini@unisi.it (L.T.); 2 CCM Italia research network (www.ccmitalia.unito.it), 10043 Torino, Italy; andrea.perrelli@unito.it (A.P.); claudia.fornelli@unito.it (C.F.); francesco.retta@unito.it (S.F.R.); 3 Department of Agriculture, Forest and Food Sciences, Plant Genetics and Breeding, University of Torino, 10095 Torino, Italy; 4 Department of Life Sciences, University of Siena, 53100 Siena, Italy; sandra.donnini@unisi.it; 5 Department of Clinical and Biological Sciences, University of Torino, 10043 Torino, Italy; giovanni.berta@unito.it (G.N.B.); federica.discipio@unito.it (F.D.S.)

**Keywords:** nutraceuticals, polyphenols, oats avenanthramides, yeast-derived recombinant avenanthramides, colon cancer cells, proliferation, migration, epithelial-mesenchymal transition (EMT), chemoprevention

## Abstract

Avenanthramides (Avns), polyphenols found exclusively in oats, are emerging as promising therapeutic candidates for the treatment of several human diseases, including colon cancer. By engineering a *Saccharomyces cerevisiae* strain, we previously produced two novel phenolic compounds, *N*-(*E*)-*p*-coumaroyl-3-hydroxyanthranilic acid (Yeast avenanthramide I, YAvnI) and *N*-(*E*)-caffeoyl-3-hydroxyanthranilic acid (Yeast avenanthramide II, YAvnII), which are endowed with a structural similarity to bioactive oat avenanthramides and stronger antioxidant properties. In this study, we evaluated the ability of these yeast-derived recombinant avenanthramides to inhibit major hallmarks of colon cancer cells, including sustained proliferation, migration and epithelial-mesenchymal transition (EMT). Using the human colon adenocarcinoma cell line HT29, we compared the impact of YAvns and natural Avns, including Avn-A and Avn-C, on colon cancer cells by performing MTT, clonogenic, adhesion, migration, and anchorage-independent growth assays, and analyzing the expression of EMT markers. We found that both YAvns and Avns were able to inhibit colon cancer cell growth by increasing the expression of p21, p27 and p53 proteins. However, YAvns resulted more effective than natural compounds in inhibiting cancer cell migration and reverting major molecular features of the EMT process, including the down-regulation of E-cadherin mRNA and protein levels.

## 1. Introduction

Avenanthramides (Avns) are low molecular weight phenolic amides (also known as *N*-cinnamoylanthranilate alkaloids or anthranilic acid amides) that are uniquely found in oats, where they act mainly as key phytoalexins in the plant defense mechanisms against certain pathogens, such as fungi [1,2]. These secondary metabolites consist of an anthranilic acid linked to a hydroxycinnamic acid with an amide bond, and are constitutively expressed in the bran and outer layers of the oat kernel [3]. Oats contain a unique group of approximately 40 different types of Avns, the most abundant being Avn-A (*N*-(4’-hydroxycinnamoyl)-5-hydroxyanthranilic acid), Avn-B (*N*-(4’-hydroxy-3’-methoxycinnamoyl)-5-hydroxyanthranilic acid), and Avn-C (*N*-(3’-4’-dihydroxycinnamoyl)-5-hydroxyanthranilic acid), which are amides of 5-hydroxyanthranilic acid with p-coumaric, ferulic, and caffeic hydroxycinnamic acids, respectively [1,4,5].

Although they are not essential nutrients, Avns have been found to possess pleiotropic bioactivities, including antioxidant, anti-inflammatory, anti-proliferative, anti-fibrotic, anti-itching, anti-atherogenic properties, with consequent major beneficial health effects. Indeed, a number of *in vitro* and *in vivo* studies suggest a protective role of Avns against several chronic diseases, such as cardiovascular diseases, cancer, and diabetes [5,6,7]. In particular, accumulated evidence demonstrate that oat Avns have the potential to reduce the risk of colon cancer and slow its progression by targeting and modulating different signaling pathways associated with colon cancer development [5,8,9].

Colon cancer is a world-wide health problem, being the second-most dangerous type of cancer with an increasing incidence and mortality trend in the last few years, and has a clear correlation with dietary habits, thus highlighting the need for multiple alternative treatment options along with effective prophylactic strategies. Accordingly, growing evidence points to the translational potential of plant-derived dietary factors known as nutraceuticals, including Avns, for the better management of colon cancer through consumption of nutraceutical-rich diets and their intervention in cancer therapeutics [8,9,10,11,12]. Indeed, dietary phenolic compounds have been shown to counteract multiple mechanisms involved in colon carcinogenesis, including tumor cell proliferation, migration, and survival, as well as tumor angiogenesis, inflammation, and metastasis [13,14]. In particular, among the pleiotropic action mechanisms that have been reported for chemopreventive phenolic nutraceuticals to retard, block, or reverse carcinogenesis, special attention is paid to the capacity of targeting critical steps of cancer metastasis, including epithelial-mesenchymal transition (EMT), an evolutionarily conserved developmental program that has been implicated in conferring metastatic properties upon epithelium-derived cancer cells by enhancing mobility, invasion, and resistance to apoptotic stimuli [15,16,17]. As metastasis is the major cause of cancer-related deaths, the prevention and treatment of the metastatic process are indeed fundamental to improving clinical outcomes. During EMT, cancer cells develop a mesenchymal phenotype where cells lose their cell-cell adhesion, cell polarity and differentiation properties by modifying the expression levels of epithelial cell adhesion proteins, such as E-cadherin, and mesenchymal proteins, such as N-cadherin or vimentin. In particular, the loss of E-cadherin expression is universally acknowledged as an important molecular hallmark of EMT; therefore, pharmacological induction of E-cadherin expression through dietary nutraceuticals represents a promising therapeutic approach for reducing the risk of colon cancer development and progression [18,19].

**Figure 1:**
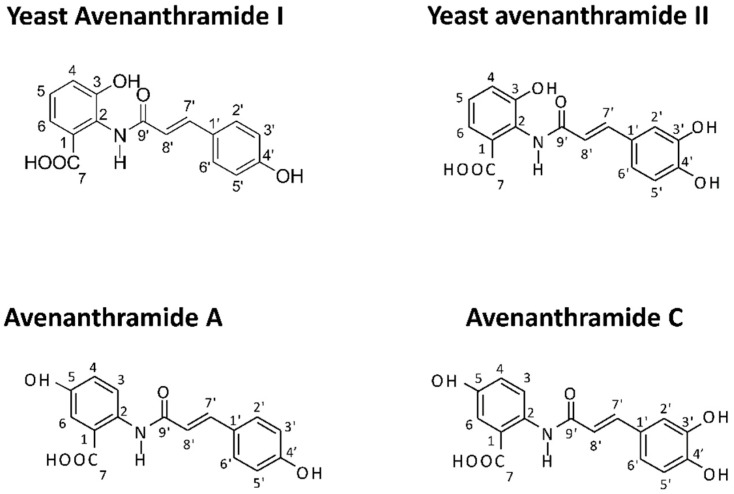
Molecular structure of yeast (YAvns) and oat avenanthramides. The structure of YAvnI and YAvnII differ from Avenathramide A (Avn-A) and Avenanthramide C (Avn-C) respectively in the position of the hydroxyl group relative to the amide bond.

Considering the wide range of potential therapeutic applications of Avns, specific efforts have been devoted to their economical and sustainable production at scales suitable for industrial applications, including novel approaches based on genetic engineering strategies as eco-friendly alternatives to conventional chemical synthesis or purification from plant sources. Indeed, knowledge of the biosynthetic pathways has now made it possible to synthesize Avns through genetically engineered microorganisms, including *Saccharomyces cerevisiae* and *Escherichia coli*, which have already allowed the biological production of a wide range of naturally occurring Avns and their analogs, helping to further elucidate and exploit the beneficial bioactivities of these emerging phenolic nutraceuticals [20,21,22,23]. In particular, by engineering a *Saccharomyces cerevisiae* strain with two plant genes (*4cl-2* from tobacco and *hct* from globe artichoke) encoding key proteins involved in the biosynthesis of phenolic esters, we have previously produced two novel yeast-derived recombinant Avns, namely *N*-(4’-hydroxycinnamoyl)-3-hydroxyanthranilic acid (YAvnI) and *N*-(3’-4’-dihydroxycinnamoyl)-3-hydroxyanthranilic acid (YAvnII) [24]. These novel Avns share structural similarity with two major oat Avns, respectively YAvnI with Avn-A and YAvnII with Avn-C, differing only in the position of the hydroxyl group in the anthranilic moiety (Figure 1) [24].

Furthermore, they were shown to possess bioactive properties relevant to biomedical applications, including a potent antioxidant activity related to their capacity of stimulating master regulators of cellular antioxidant responses [24,25], as well as putative anti-inflammatory and antiproliferative properties related to their capacity of inhibiting nuclear factor-kappa B (NF-κB) activation [26] and cyclin D1 expression [25].

In the present study, we compared the impact of yeast-derived recombinant Avns, YAvnI and II, and major natural Avns, Avn-A and Avn-C, on colon cancer cells, showing that YAvns were as effective as natural Avns in inhibiting tumor cell growth and survival, but were more effective in inhibiting EMT and reducing tumor cell migration through transcriptional regulation of E-cadherin, suggesting enhanced functional and nutraceutical properties that might be advantageous with respect to colon cancer management and metastasis prevention.

## 2. Materials and Methods 

### 2.1. Cell Lines

The HT29 and WiDr human colorectal adenocarcinoma cell lines, and the CCD-18Co (ATCC^®^ CRL-1459^™^) human fibroblast cell line isolated from normal colon tissue (both from American Type Culture Collection, Manassas, VA, USA) were cultured in 75 cm^2^ flasks (TPP AG, Trasadingen, Switzerland) in RPMI-1640 (Euroclone, Milano, Italy) supplemented with 10% (*v*/*v*) fetal calf serum (FCS) (Euroclone, Milano, Italy), 100 U/mL penicillin G, 40 µg/mL gentamycin sulfate, and 2.5 µg/mL amphotericin B (all from Sigma-Aldrich, Milano, Italy) at 37 °C in a humidified 5% CO_2_ atmosphere. Cells were harvested twice a week with 0.25% porcine trypsin solution (Sigma-Aldrich, Milano, Italy). 

### 2.2. Chemicals

Stock solutions of YAvnI and YAvnII, obtained as previously described [25], and Avn-C and Avn-A (Sigma-Aldrich, Milano, Italy) were prepared by dissolving compounds in dimethyl sulfoxide (DMSO) (Sigma-Aldrich, Milano, Italy) to a final concentration of 0.1 M. 

### 2.3. MTT Cell Viability Assay 

The MTT-based colorimetric assay for the nonradioactive quantification of cell viability was performed as previously described [27]. This assay is especially suitable as a sensitive and reliable indicator of the cellular metabolic activity, and it is therefore useful to assess the effects of cell treatments in short time intervals (i.e., 1–3 days). Specifically, it relies on the reduction of the water-soluble tetrazolium dye MTT (3-(4,5-dimethylthiazol-2-yl)-2,5-diphenyltetrazolium bromide) into an insoluble colored formazan product primarily by the mitochondrial dehydrogenases of viable cells, thus evaluating mainly the metabolic state/viability of all cells in culture by virtue of a linear relationship between cell activity/viability and absorbance of the formazan product. Briefly, cells were seeded at the density of 2500 cells/well in 96 multiwell plates in RPMI-1640 medium supplemented with 10% serum. After adhesion, cells were treated with either Avn-A, Avn-C, YAvnI or YAvnII (50, 100, 200 μM) for 68 h. The medium was then removed, and cells were incubated for 4 h with fresh medium in the presence of 1.2 mM MTT (3-(4,5-dimethylthiazol-2-yl)-2,5-diphenyltetrazolium bromide) (Sigma-Aldrich, Milano, Italy), which is cleaved by viable cells to generate the corresponding pigmented formazan product, whose amount directly correlates to the number of metabolically active cells in the culture. Afterward, the medium was removed, 100 microliter of DMSO was added to each well to dissolve the blue formazan crystals, and the plates were incubated at 37 °C for 10 min. The absorbance of the formazan dye was then measured at 570 nm with a microplate reader (VersaMax Tunable Microplate Reader, Molecular Devices, San Jose, CA, USA), and used to calculate cell viability. Data were expressed as a percentage of the basal control.

### 2.4. Clonogenic Assay

The clonogenic assay is useful for testing the proliferative capability of cultured cells by virtue of the cell’s ability to undergo sufficient proliferation to form a colony, thus being especially suitable for assessing long-term effects of cell treatments (e.g., a few weeks after treatment). In particular, as the cell metabolic state and clonogenic potential are not necessarily parallel events, the clonogenic assay can add valuable and distinct information to that provided by the MTT assay and may be seen as complementary. Briefly, cells were plated in 6 multi-well plates (700 cells/dish) in medium containing 10% FCS (Euroclone, Milano, Italy). After 24 h cells were treated with Avn-A, Avn-C, YAvnI and YAvnII (200 μM) or DMSO as a control, in 10% FCS (*v*/*v*), and kept in a humidified incubator for 10 days. Colonies (>50 cells) were fixed and stained with Diff-Quik, counted and photographed.

### 2.5. Protein Extracts and Western Blot Analysis

Cells were seeded at the density of 350,000 cells/well in 6 multi-well plates in medium with 10% serum. After adhesion, cells were treated for 48 h with either Avn-A, Avn-C, YAvnI or YAvnII (200 μM). Cell lysates were then obtained and analyzed as previously described [28]. Briefly, cell lysates were centrifuged at 15,000×·*g* for 20 min at 4 °C, and equal amounts of protein extracts were analyzed by polyacrylamide gel electrophoresis and Western blotting onto activated nitrocellulose membranes. Unspecific protein-binding sites were blocked by incubation with 5% milk 0.5% Tween-20 in Tris-buffered saline (TBS) for 1 h at room temperature, and membranes were then incubated overnight at 4 °C with appropriate dilutions of specific primary antibodies. Membranes were then washed with TBS 0.1% Tween-20, and incubated for 1h at room temperature with horseradish peroxidase-conjugated anti-mouse (1:2500) (Promega, Milano, Italy) or anti-rabbit (1:10.000) (Merck Millipore, Milano, Italy) secondary antibody, followed by enhanced chemiluminescence detection system (Bio-Rad, Milano, Italy). As an internal control for protein loading, membranes were re-probed with antibodies for housekeeping proteins, including β-actin and glyceraldehyde 3-phosphate dehydrogenase (GAPDH). Images were finally digitalized with Image Quant LAS4000 (GE Healthcare Europe GmbH, Milano, Italy). Quantitative determination of immunoreactive bands was performed by densitometry using the ImageJ software (open source image processing program, National Institutes of Health, Bethesda, MD, USA), and data were normalized to the levels of internal control. 

Primary antibodies used in the present study included: anti p27 (1:1000) (Cell Signaling, Euroclone, Milano, Italy), anti p21 (1:1000) (Cell Signaling, Euroclone, Milano, Italy), anti p53 (1:1000) (Santa Cruz, Heidelberg, Germany), anti-focal adhesion kinase (1:1000) (Santa Cruz, Heidelberg, Germany), anti P-FAK (1:1000) (Merck Millipore, Milano, Italy), anti E-cadherin (1:1000) (Cell Signaling, Euroclone, Milano, Italy), anti β-actin (1:1000) (Sigma-Aldrich, Milano, Italy), anti GAPDH (1:10,000) (Merck Millipore, Milano, Italy). 

### 2.6. Immunofluorescence Analysis

Cells (5 × 10^4^ cells/well on glass cover-slips placed into 24 multi-well plates) were maintained in 10% FCS for 24 h. Cells were then treated with Avn-A, Avn-C, YAvnI and YAvnII (200 μM, 48 h) and fixed in acetone for 5 min. After blocking of unspecific bindings with 3% bovine serum albumin (BSA), cells were incubated overnight at 4 °C with the primary antibody (anti FAK, 1:80). Samples were then incubated with a secondary antibody Alexafluor 488, and analyzed by confocal microscopy (Zeiss LSM700) at 60× magnification.

### 2.7. Adhesion Assay

Cells were maintained in 10% FCS and then trypsinized; 5 × 10^4^ cells/mL in 1% FCS medium were seeded in 96 multiwell plates and incubated for 2 h at 37 °C in presence of Avn-A, Avn-C, YAvnI and YAvnII (200 μM) or DMSO as a control. The wells were washed gently with phosphate-buffered saline (PBS) (Sigma-Aldrich, Milano, Italy), and adherent cells were fixed and stained with Diff-Quik. Adherent cells were counted in five randomly selected microscopic fields at 200× total magnification.

### 2.8. Anchorage-Independent Cell Viability Assay

Cells at a density of 5 × 10^5^ cells/mL were incubated in suspension in medium with 0.1% FCS for 48 h in presence of either Avn-A, Avn-C, YAvnI or YAvnII (200 μM), or DMSO as a control. After incubation, the ratio of dead cells stained with trypan blue versus total cells was evaluated by light microscopy. The number of dead cells was reported as a percentage of total cells.

### 2.9. Migration Assay

Chemotaxis experiments were performed with the Boyden chamber technique. 1.25 × 10^4^ HT29 cells were added to the upper wells of the chamber and Avn-A, Avn-C, YAvnI and YAvnII (200 μM) or DMSO as a control were added in the lower wells of the chamber in medium with 10% FCS. Migration was measured in triplicate by counting the number of cells that had moved in 18 h across the filter coated with gelatin (1%). Cells were counted in five random fields/well at a magnification of 40×. Data are reported as total number of counted cells/well.

### 2.10. Real-Time PCR

Cells were seeded at the density of 350,000 cells/well in 6 multiwell plates in medium with 10% serum. After adhesion, cells were treated for 48 h with either Avn-A, Avn-C, YAvnI or YAvnII (200 μM). Cells were then lysed, and total RNA was obtained using an RNA Mini kit (Qiagen, Milano, Italy). cDNAs were synthesized from 1.0 μg total RNA using a High Capacity RNA-to-cDNA Kit (Thermo Fisher Scientific, Milano, Italy) according to the manufacturer’s instructions. Primers (Table 1) were designed on the basis of the coding sequence (CDS) using the Primer 3 software (http://bioinfo.ut.ee/primer3-0.4.0/). As a housekeeping gene, GAPDH was chosen for its stability and level of expression, which is comparable to the genes of interest and whose expression remained stable after cell treatment.

PCR reactions were carried out in 96-well optical plates using the StepOnePlus Real-Time PCR System and the Power SYBR Green PCR Master Mix (Thermo Fisher Scientific, Milano, Italy). Three biological replicates for each tested sample were performed in a reaction volume of 20 L. The PCR conditions comprised an initial incubation of 95 °C/10 min, followed by 35 cycles of 95 °C/15 s and 60 °C/1 min. In all experiments, appropriate negative controls containing no template were subjected to the same procedure to detect or exclude any possible contamination. Melting curve analysis was performed at the end of amplification. Amplicons were analyzed by the comparative threshold cycle method, in which ΔΔCt is calculated as ΔCtI-ΔCtM, where ΔCtI is the Ct value for the any target gene normalized to the endogenous housekeeping gene and ΔCtM is the Ct value for the calibrator, which is also normalized to housekeeping gene. 

### 2.11. Statistical Analysis

Data were generated from three independent experiments and expressed as means ± standard deviation (SD). Statistical significance, determined by Student’s *t*-test, was set at *p* < 0.05.

## 3. Results

### 3.1. Effects of Natural and Yeast-Derived Recombinant Avenanthramides on Cancer Cell Viability

**Figure 2:**
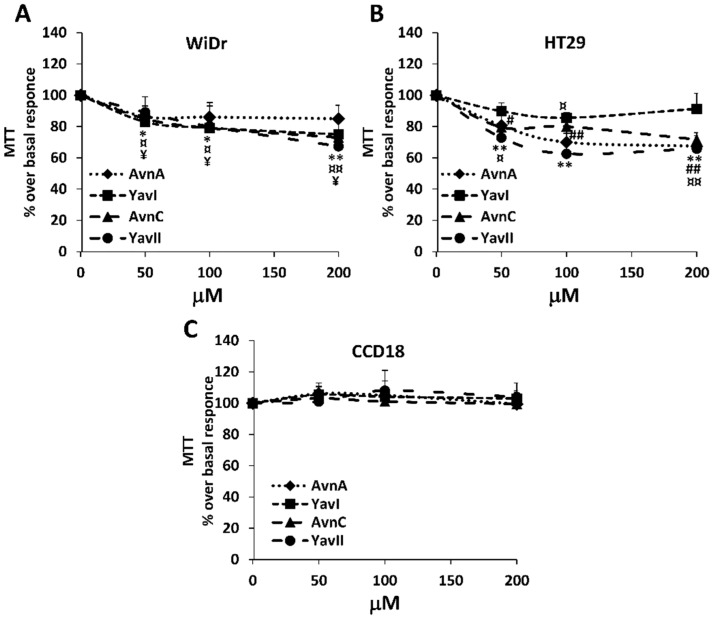
Effects of natural and yeast derived avenanthramides on cancer cell viability. Cells viability was evaluated by the MTT assay. Cells were exposed to increasing concentration of Avns and YAvns (50, 100 and 200 μM) for 3 days. Data are expressed as percentage over basal control. Statistical analysis: AvnA: ^##^ *p* < 0.01 and ^#^ *p* < 0.05; YavI: ^¥^ *p* < 0.05; AvnC: ^¤¤^ *p* < 0.01 and ^¤^ *p* < 0.05; YavII: ** *p* < 0.01 and *p < 0.05 vs. basal control.

To evaluate the effects of natural and yeast-derived recombinant avenanthramides on the viability of colon cancer cells, we treated HT29 and WiDr human colorectal adenocarcinoma cells with different concentrations of either Avns or YAvns (from 50 to 200 μM) and performed the MTT assay at 72 h. Normal colon fibroblasts (CCD18) were used as control. As shown in Figure 2, neither of the compounds tested modified the viability of CCD18 cells (Figure 2C). However, a dose-dependent sensitivity to all compounds tested was seen in HT29 and WiDr cells, with a statistically significant decrease in cell viability observed in both colon cancer cells treated with the higher doses of Avn-C and YAvnII (100–200 µM) (Figure 2A,B), and a higher sensitivity of HT29 cells at the lower doses (50–100 µM) (Figure 2B), suggesting that both Avns and YAvns can exert selective inhibitory effects on the viability of colon cancer cell lines, as compared to normal colon cells. Even though the MTT assay does not distinguish between decreased levels of proliferation or increased cytotoxicity, previous evidence demonstrates that Avns can both inhibit cell proliferation and stimulate apoptosis [9,25,29]. In this light, the reduced number of viable cells observed in colon cancer cell lines, with respect to normal colon cells, upon treatment with oat Avns and YAvns might be attributable to either a decreased cell proliferation or an increased cytotoxicity, or indeed, both.

### 3.2. Natural and Yeast-Derived Avenanthramides Reduce Growth of HT29 Cancer Cells

In light of the outcomes of the MTT assay, we used HT29 cells to comparatively investigate the potential anticancer properties and mechanisms of action of natural and yeast-derived Avns.

**Figure 3:**
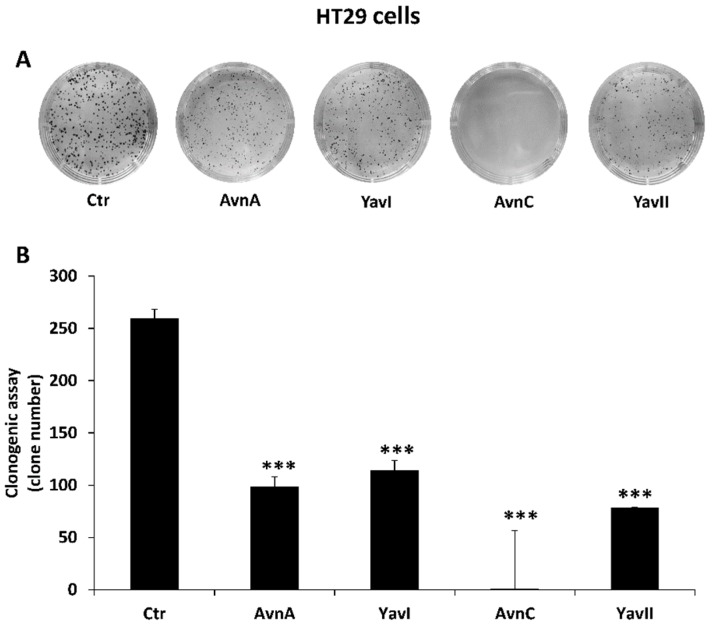
Natural and yeast-derived avenanthramides reduce clonogenicity of HT29 colon cancer cells. (**A**) Representative images of colony formation capability of HT29 cells in response to Avn-A, Avn-C, YAvnI and YAvnII (200 μM) for 10 days; (**B**) Number of colonies in HT29 cells in response to natural or yeast derived avenanthramides over control. ***p < 0.001.

Firstly, we performed clonogenic assays to compare the efficacy of Avns and YAvns for inhibiting colon cancer cell growth and colony formation. To this end, HT29 cells were incubated with either Avn-A, Avn-C, YAvnI, or YAvnII at the same concentration (200 μM) for 10 days. Experimental outcomes showed that both natural and yeast-derived recombinant avenanthamides were able to significantly restrain the colony formation of HT29 cells (Figure 3A,B), implying that both Avns and YAvns are indeed effective in inhibiting proliferation and colony formation ability of colon cancer cells.

**Figure 4:**
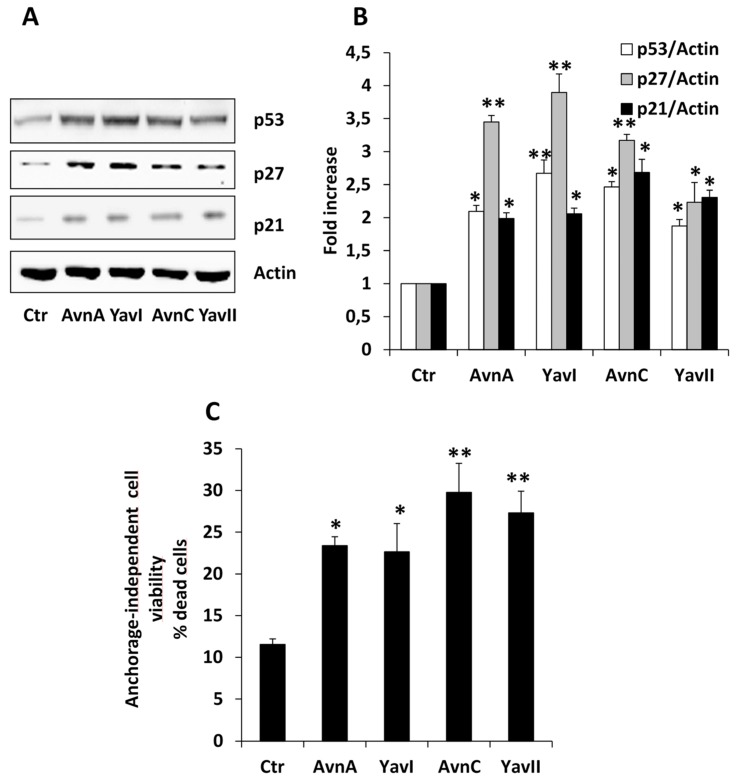
Induction of cell cycle blocking proteins and apoptosis by Avns and YAvns. (**A**,**B**) Representative images and quantification of western blot analysis of p53, p27 and p21 expression in HT29 cells treated with Avn-A, YAvnI. Avn-C and YAvnII (200 μM, 48 h). * *p* < 0.05 and ** *p* < 0.01 and vs. Ctr; (**C**) Cell vitality of HT29 in suspension treated with either Avn-A, YAvnI, Avn-C or YAvnII (200 μM, 48 h) in 0.1% of serum. Results are expressed as % of dead cells. * *p* < 0.05 and ** *p* < 0.01 vs. Ctr.

Previous studies showed that the inhibitory effects of Avns on cell proliferation may be attributed to their ability to modulate the expression levels of important regulators of cell cycle progression, including the downregulation of cyclin D1 [25,29], and the upregulation of p21 and p27 cyclin dependent kinase inhibitors and their major regulator p53 [30]. In this light, and considering our previous demonstration that both Avns and YAvs were indeed able to inhibit the expression cyclin D1 [25], we then analyzed comparatively the effects of YAvs and Avns on the expression of the cell cycle inhibitor proteins p53, p21 and p27. As shown in Figure 4A,B, the levels of these proteins were significantly enhanced upon treatment of HT29 cells with Avn-A, Avn-C, YAvnI and YAvnII (200 μM for 48 h), demonstrating that both Avns and YAvs exert anti-proliferative effects on HT29 cancer cells by modulating the expression of major cell cycle regulatory proteins.

Previous work has characterized the process of anchorage-independent growth of cancer cells in vitro as a key aspect of the tumor phenotype, particularly with respect to metastatic potential [31,32]. To further investigate comparatively the putative anticancer properties of Avns and YAvns, we then analyzed the effect of these compounds on the vitality of HT29 cells cultured in suspension condition, demonstrating that treatment with 200 μM of either Avn-A, Avn-C, YAvnI, or YAvnII for 48 h caused a marked increase in the number of dead HT29 cells (Figure 4C).

### 3.3. Yeast-Derived Recombinant Avenanthramides Exhibit Superior Anti-Migratory and Anti-EMT Activities

**Figure 5:**
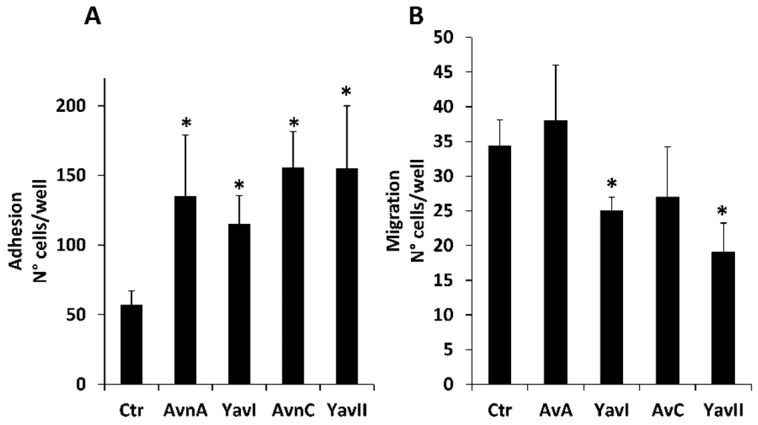
Role of natural and yeast derived avenanthramides on tumor cell adhesion and migration. (**A**) Adhesion of HT29 cells untreated or treated with Avn-A, Avn-C, YAvnI and YAvnII (200 μM) on 96 well plate. Cell adhesion was evaluated after 2 h of incubation in 1% serum. Results (three experiments in triplicate) are expressed as number of adherent cells. * *p* < 0.05; (**B**) HT29 were evaluated for their chemotactic effect toward Avn-A, Avn-C, YAvnI and YAvnII (200 μM) in 10% serum after 18 h of incubation. Data are reported as number of migrated cells counted per well. * *p* < 0.05 vs. basal control.

It is well known that normal cells die through apoptosis when detached from extracellular matrix. However, cancer cells undergo phenotypic changes, acquiring the ability to survive and grow under anchorage-independent conditions, as well as to leave the original tumor site, migrate through surrounding tissues and establish metastasis to a distant site. To compare the capacity of natural and yeast-derived recombinant Avns to reduce the metastatic potential of colon cancer cells, we first analyzed their effects on adhesion and migration of HT29 cells. As shown in Figure 5A, both Avns (Avn-A and Avn-C) and YAvns (YAvnI and YAvnII) were effective in increasing adhesion of treated HT29 cells with respect to untreated cells (Figure 5A). However, only YAvnI and YAvnII were able to significantly reduce HT29 cell migration (Figure 5B), suggesting that these compounds possess an enhanced anti-migratory activity on colon cancer cells.

**Figure 6:**
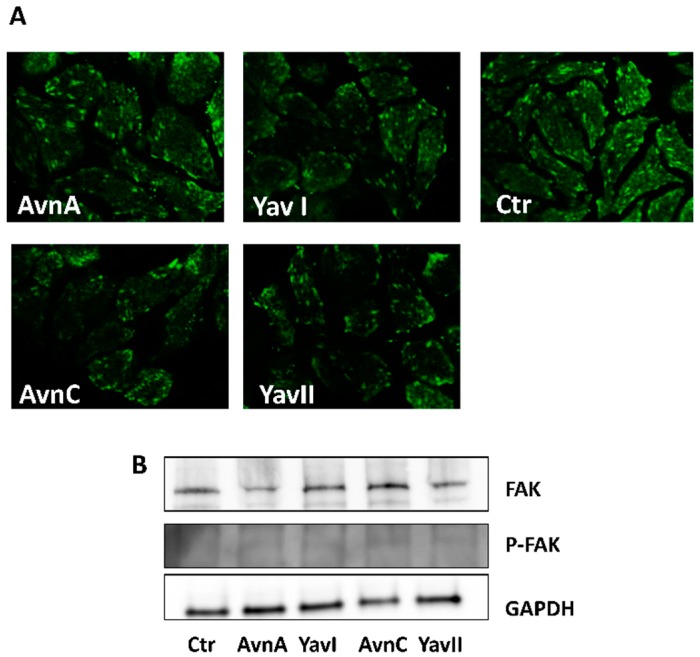
Effects of natural and yeast-derived avenanthramides on focal adhesion kinase (FAK) expression and phosphorylation. (**A**) Representative images of immunofluorescence analysis of FAK in HT29 cells treated with either Avn-A, YAvnI, Avn-C or YAvnII (200 μM, 48 h); (**B**) Western blot analysis of FAK expression and phosphorylation (P-FAK) levels in HT29 cells treated with either Avn-A, YAvnI, Avn-C or YAvnII (200 μM) for 48 h. Images are representative of three different experiments.

In the attempt to identify the molecular mechanisms underlying the superior efficacy of YAvns in reducing HT29 cell migration, we analyzed the expression and activation of focal adhesion kinase (FAK), a protein known to promote malignancy, by regulating the metastatic potential of cancer cells through highly-coordinated signaling networks that drive cell migration and tissue invasion [33]. The HT29 cells were treated for 48 h with either Avn-A, Avn-C, YAvnI or YAvnII (200 μM) and then analyzed by immunofluorescence and western blotting. As shown in Figure 6A,B, neither FAK subcellular localization nor its expression and phosphorylation/activation levels were affected by cell treatment with either Avns or YAvns, suggesting that the superior anti-migratory activity of YAvns does not involve the modulation of FAK.

**Figure 7:**
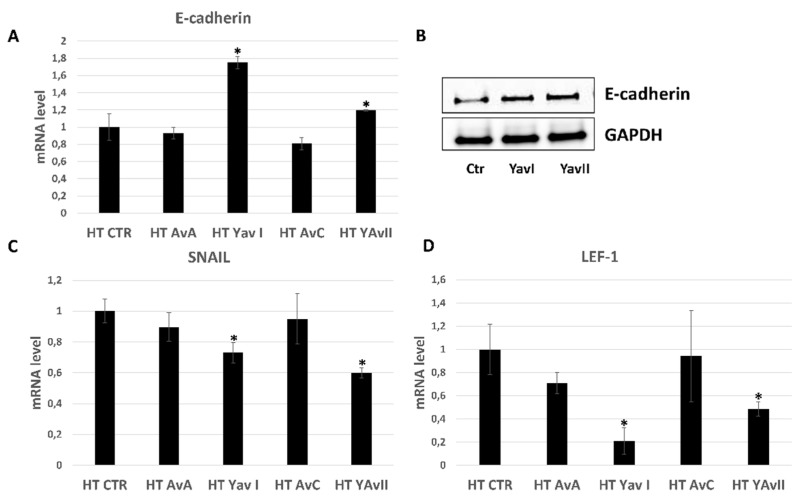
Yeast avenanthramides regulate E-cadherin expression. (**A**) Quantitative PCR for E-cadherin mRNA expression in cells exposed to YAvnI and YAvnII (200 μM, 48 h). E-cadherin mRNA expression is reported as fold increase compared with basal levels (* *p* < 0.05); (**B**) Representative images of western blot analysis of HT29 cells exposed to YAvnI and YAvnII (200 μM, 48 h); (**C**,**D**) Quantitative PCR for Snail1 and LEF-1 expression in cells exposed to YAvnI and YAvnII (200 μM, 48 h). Data are expressed as fold increase compared with control (* *p* < 0.05).

Given the important role of E-cadherin downregulation and epithelial-mesenchymal transition (EMT) in phenotypic changes underlying the acquired enhanced motility and invasion of epithelium-derived cancer cells [15,16,17,18], we analyzed the expression levels of E-cadherin on HT29 cells after 48 h treatment with natural and yeast-derived recombinant avenanthramides. Real time PCR experiments showed that only YAvnI and YAvnII increased E-cadherin mRNA levels by 1.72 and 1.2 fold, respectively, as compared with the control (Figure 7A). These data were confirmed by western blot analysis, which showed an upregulation of E-cadherin protein levels upon cell treatment with YAvns (Figure 7B). Furthermore, they correlated with the observed enhanced inhibitory effects of YAvns on HT29 cell migration (Figure 5B), suggesting a potential relationship. To assess whether the capacity of YAvns to modulate E-cadherin expression levels involved transcriptional regulation, we analyzed the mRNA levels of Snail1 and lymphoid-enhancing factor 1 (LEF-1) transcription factors upon treatment of HT29 cells with either Avn-A, Avn-C, YAvnI or YAvnII, showing that only YAvns were effective in reducing Snail1 and LEF-1 mRNA expression (Figure 7C,D), and suggesting that the modulation of E-cadherin expression levels by YAvns occur through transcriptional regulation. 

Overall, our data demonstrate the superior capacity of YAnvs in inhibiting EMT and migratory processes in colon cancer cells, suggesting promising activities against the metastatic potential of colorectal cancer cells that deserve further study using in vivo models.

## 4. Discussion

Colorectal cancer (CRC) incidence and mortality rates are rising rapidly in many countries worldwide, often reflecting the adoption of western diets and lifestyles. Multiple interventions, including prophylactic and therapeutic approaches through dietary nutraceuticals, are therefore needed to reduce the number of patients with CRC in future decades [10,11,12,34,35]. Accordingly, growing evidence clearly demonstrates the medicinal importance of nutraceuticals and their ability to reduce the risk, and retard, block, or reverse the progression of colon cancer by targeting and modulating different signaling pathways involved in carcinogenesis through pleiotropic mechanisms of action [10,12,34,35].

Among nutraceuticals with potential medicinal benefits, avenanthramides (Avns), phenolic compounds found exclusively in oats, have growingly become promising as potential therapeutic candidates for the treatment of several important human diseases, such as cancer, diabetes and cardiovascular diseases [5,6,7,8,9]. Indeed, Avns have been found to be bioavailable in humans, and shown to possess major bioactivities, including antioxidant, anti-inflammatory, and anti-proliferative activities, which are related to their capacity to influence multiple molecular mechanisms [5,6,8,36,37]. Besides natural Avns isolated from oats and correspondent synthetic compounds [38,39], novel Avn analogs endowed with important bioactivities have been produced through genetically engineered microorganisms, including Tranilast^TM^ (*N*-[3′,4′-dimethoxycinnamoyl]-anthranilic acid), a pharmaceutical drug currently used in Japan and South Korea to treat allergic disorders [20,40]. Indeed, biological production of valuable Avns by genetically engineered microorganisms has several advantages over direct extraction and purification from plant sources or conventional chemical synthesis, such as fewer requirements for toxic chemicals and natural sources, simple extraction, consistent quality, and economical and sustainable production.

In light of this, we previously developed a genetic engineering strategy for producing novel phenolic compounds in *Saccharomyces cerevisiae*, generating two novel Avn analogs, YAvn I and YAvn II, which showed structural similarity to Avn-A and Avn-C, respectively, and potent antioxidant properties [24]. Furthermore, they were effective in rescuing major phenotypic hallmarks of Cerebral Cavernous Malformation (CCM) disease, a cerebrovascular disorder of genetic origin that has been linked to oxidative stress [25,26,37]. To further characterize the biological properties of the two yeast-derived recombinant Avns, herein we investigated their effects on colon cancer cells, as compared with those of major natural oat Avns, including Avn-A and Avn-C. Indeed, while the inhibitory effects of some oat Avns on human colon cancer cell lines have been previously reported [9], structure-activity relationship analyses have clearly demonstrated that Avn properties are dictated by the number and position of the hydroxyl groups, and the nature of substitutions on the aromatic rings [38,41,42], suggesting that even slight structural variations may account for significant differences in the biological activities of distinct yeast-derived and oat Avns. Therefore, using the human colon adenocarcinoma cell line HT29 as experimental model, we compared the impact of YAvns and natural oat Avns on colon cancer cells by performing MTT, clonogenic, adhesion, migration, and anchorage-independent growth assays, and analyzing the expression of important proteins involved in colon cancer cell proliferation and metastasis, including key regulators and markers of cell cycle progression and epithelial-mesenchymal transition (EMT).

Our experimental outcomes showed that YAvns were as effective as natural Avns in inhibiting colon cancer cell proliferation, clonogenicity and anchorage-independent growth, and enhancing cell adhesion, as well as in increasing the expression of major cell cycle regulators, such as p21, p27 and p53 proteins, suggesting that YAvns and natural Avns share a common capacity in preventing and reversing key events involved in cancer development and progression. Consistent with these findings, whereas there is evidence in vascular smooth muscle cells that the inhibition of cell proliferation promoted by Avns implicates the upregulation of the p53–p21 pathway [30], we previously reported that YAvns can promote the downregulaton of cyclin D1 required for cell transition from proliferative growth to quiescence [25]. Noteworthy, apart from many functional similarities between YAvns and natural Avns, we found that YAvns were more effective than the natural compounds tested in inhibiting colon cancer cell migration and reverting major molecular features of the EMT process, including the downregulation of E-cadherin mRNA and protein levels, and the upregulation of the transcription factors Snail1 and LEF-1, suggesting that the peculiar molecular structure of YAvns endows them with an enhanced potential capacity to limit EMT-mediated cancer progression and metastasis. Accordingly, besides playing a major physiological role in normal development, the conversion of cells from an epithelial, adhesive state to a mesenchymal, motile state mediated by the pathological activation of molecular mechanisms underlying the EMT process is a key event in the development of cancer metastasis [43,44], and it has consequently received significant interest as an important target in cancer prevention and treatment [18,44,45,46]. Consistent with our findings, there is indeed emerging evidence that a number of phytochemicals, including phenolic nutraceuticals, can reduce the metastatic potential of cancer cells by inhibiting EMT pathways [18,45,46,47]. In particular, the anti-EMT activities of dietary phytochemicals have been related to their ability to counteract the loss of E-cadherin expression, a major EMT hallmark that has been directly correlated to the development of metastatic cancers [48]. Remarkably, as further support to our findings, there is also evidence that the upregulation of E-cadherin expression promoted by phytochemicals may involve the downregulation of transcription factors that suppress E-cadherin gene expression and facilitate the transition from epithelial to mesenchymal state, such as Snail1 and LEF-1 [18,46,48,49].

Interestingly, the enhanced anti-migratory and anti-EMT properties of YAns vs. natural compounds, including Avn-A and Avn-C, might be related to differences in the substitution patterns of the two aromatic moieties, including the hydroxyanthranilic acid moiety (A ring) and the hydroxycinnamic acid moiety (B ring) (Figure 1). Indeed, while the A ring of major natural Avns, such as Avn-A and Avn-C, consists of 5-hydroxyanthranilic acid, the A ring of YAvns, including YAvnI and YAvnII, consist of 3-hydroxyanthranilic acid, which might confer to these compounds the peculiar properties identified in this study. Consistently, previous structure-function relationship studies attributed specific functions to the two aromatic moieties of distinct Avns [41,42,50]. On the other hand, while the in vivo bioavailability and effective concentrations of natural Avns have been previously assessed [3,5,36], the bioavailability and effective concentrations in vivo of yeast-derived Avns are currently unknown and need to be investigated by specific studies in animal models, including available mouse models of colorectal cancer [51].

## 5. Conclusions

Overall, this study demonstrates that YAvns possess significant anti-proliferative activity against colon cancer cells, and may exert superior anti-migratory and anti-EMT activities as compared to major natural Avns, including the ability to modulate transcriptional factors involved in E-cadherin downregulation and EMT progression, suggesting enhanced functional properties as bioactive nutraceuticals. Taken together with the feasibility and reliability of producing such bioactive nutraceuticals through the yeast-based system, their anti-proliferative activity, shared with natural Avns, and their superior anti-migratory and anti-EMT activities might be highly advantageous for colon cancer management and metastasis prevention, thus paving the way for further studies in *in vivo* models of colorectal cancer.

## Figures and Tables

**Table 1:** List of primers used for Real-Time PCR analyses.

Genes	Primer Forward	Primer Reverse
**GAPDH**	5′-TGCACCACCAACTGCTTAGC	5′-GGCATGGACTGTGGTCATGAG
**LEF-1**	5′-CCACGGACGAGATGATCCCC	5′-GCTGGCCGGGATGATTTCAG
**Snail**	5′-ATGCCGCGCTCTTTCCTCGTC	5′-AGCAGGTGGGCCTGGTCGTAG
**E-cadherin**	5′-AATATGTTCACCATTAACAG	5′-GTATACGTAGGGAAACTCTC

## Abbreviations

Avn	Avenanthramide
CCM	Cerebral cavernous malformations
CRC	Colorectal cancer
EMT	Epithelial-mesenchymal transition
YAvn	Yeast avenanthramide
